# The Influence of Calcium Glycerophosphate (GPCa) Modifier on Physicochemical, Mechanical, and Biological Performance of Polyurethanes Applicable as Biomaterials for Bone Tissue Scaffolds Fabrication

**DOI:** 10.3390/polym9080329

**Published:** 2017-08-01

**Authors:** Justyna Kucińska-Lipka, Iga Gubanska, Olexandr Korchynskyi, Khrystyna Malysheva, Marcin Kostrzewa, Damian Włodarczyk, Jakub Karczewski, Helena Janik

**Affiliations:** 1 Department of Polymer Technology, Faculty of Chemistry, Gdansk University of Technology, Narutowicza St. 11/12, 80-233 Gdansk, Poland; iga.gubanska@gmail.com (I.G.); heljanik@pg.gda.pl (H.J.); 2 Institute of Cell Biology, National Academy Science of Ukraine, 14/16 Drahomanov Str., 79005 Lviv, Ukraine; olexkor@hotmail.com (O.K.); khrystyna.malysheva@gmail.com (K.M.); 3 Centre for Innovative Research in Medical and Natural Sciences, Rzeszow University and Medical Faculty, 35-959 Rzeszow, Poland; 4 Department of Organic Materials Technology, Technical University of Radom, 26-600 Radom, Poland; m.kostrzewa@uthrad.pl; 5 Institute of Physics, Polish Academy of Science, Division of Physics and Technology of Wide-Band-Gap Semiconductor Nanostructures, Al. Lotnikow 32/46, 02-668 Warsaw, Poland; iceni@wp.pl; 6 Faculty of Applied Physics and Mathematics, Gdansk University of Technology, Narutowicza St. 11/12, 80-233 Gdansk, Poland; jkarczew@mif.pg.gda.pl

**Keywords:** polyurethane, bone tissue engineering, calcium glycerolphosphate salt, mechanical properties, contact angle, SEM, EDX, calcification, solvent casting/particulate leaching, TIPS

## Abstract

In this paper we describe the synthesis of poly(ester ether urethane)s (PEEURs) by using selected raw materials to reach a biocompatible polyurethane (PU) for biomedical applications. PEEURs were synthesized by using aliphatic 1,6-hexamethylene diisocyanate (HDI), poly(ethylene glycol) (PEG), α,ω-dihydroxy(ethylene-butylene adipate) (Polios), 1,4-butanediol (BDO) as a chain extender and calcium glycerolphosphate salt (GPCa) as a modifier used to stimulate bone tissue regeneration. The obtained unmodified (PURs) and modified with GPCa (PURs-M) PEEURs were studied by various techniques. It was confirmed that urethane prepolymer reacts with GPCa modifier. Further analysis of the obtained PURs and PURs-M by Fourier transform infrared (FTIR) and Raman spectroscopy revealed the chemical composition typical for PUs by the confirmed presence of urethane bonds. Moreover, the FTIR and Raman spectra indicated that GPCa was incorporated into the main PU chain at least at one-side. The scanning electron microscopy (SEM) analysis of the PURs-M surface was in good agreement with the FTIR and Raman analysis due to the fact that inclusions were observed only at 20% of its surface, which were related to the non-reacted GPCa enclosed in the PUR matrix as filler. Further studies of hydrophilicity, mechanical properties, biocompatibility, short term-interactions, and calcification study lead to the final conclusion that the obtained PURs-M may by suitable candidate material for further scaffold fabrication. Scaffolds were prepared by the solvent casting/particulate leaching technique (SC/PL) combined with thermally-induced phase separation (TIPS). Such porous scaffolds had satisfactory pore sizes (36–100 μm) and porosity (77–82%) so as to be considered as suitable templates for bone tissue regeneration.

## 1. Introduction

Tissue scaffolds, designed for tissues regeneration, are three-dimensional porous structures, which serve as biological tissue substitutes that enable the functional performance of the regenerated tissue to be restored, maintained or improved [1]. Tissue engineering (TE) applies both natural and synthetic polymers [2], metals [3], ceramics [4] and bioactive glasses [5]. Biomaterials used for the purpose of tissue engineering (TE) have to meet strict requirements such as biocompatibility. Moreover, these materials may reveal some bioactive behavior, which could stimulate proper tissue regeneration [6]. The 3D scaffold has to provide an adequate support for the regenerated tissue, thus the mechanical characteristics of the used biomaterials are an important feature. In addition the gradual degradation of the scaffold is necessary for proper tissue restoration [7,8]. However, in order to meet the requirements of an ideal tissue scaffold material and to combine the best mechanical properties with bioactivity and ability to degrade in the human body environment, new composite materials, which are the combination of polymers with different types of fillers, are being developed [1,8]. Polyurethanes (PU) according to their superior characteristics of proper physicochemical, mechanical, and biological properties seem to meet all of these requirements for use in TE as materials for scaffold fabrication [1,6].

PUs have been widely developed in the field of biomedical devices, thus their modification is well known [1,6,9]. The most common PU modifications for bone tissue engineering take place mainly by the introduction of the filler into the PU matrix. The most often reported fillers are calcium phosphates like hydroxyapatite [4,10,11], nanohydroxyapatite [12,13], and β-tricalcium phosphate [14,15]. Recently anew solution for PU modification was proposed in the form of calcium glycerophosphate [16], bioactive glass [5] or carbon nanotubes [17].

Calcium glycerophosphate is the calcium salt of glycerophosphoric acid. This compound has been approved for use by the Ministry of Health [18] as a nutrient, a component of dietary supplements or mineral food products, and has been considered as a safe ingredient/food additive by the US Food and Drug Administration [19]. In addition to the previously listed applications, calcium glycerophosphate is also used as an ingredient in toothpaste [20], dental varnishes [21], as well as electrolytes used for mineralization of hydrogels for bone regeneration [22] or surface modification of titanium bone implants [3].

There are several examples in the literature, describing the synthesis of PU composites with the use of fillers mentioned above leading to the desired changes in mechanical properties of the material, as well as contributing to its bioactivity improvement [4,10,11,12,13,14,15,16,17].The effect of calcium glycerolphosphate salt (GPCa) as a filler on tissue scaffolds properties which received biodegradable polyurethane foams for bone graft substitutes was recognized by Gorna et al. [16]. The PU system studied by Gorna et al. was derived from 1,6-hexamethylene diisocyanate, poly(ethylene oxide)diol, poly(ε-caprolactone)diol, amine-based polyol or sucrose-based polyol, water as a chain extender and foaming agent, catalysts, citric acid as a calcium complexing agent, lecithin or solutions of vitamin D_3_ as surfactants, and various inorganic fillers. One of the fillers used was GPCa, others were calcium carbonate and hydroxyapatite [16]. Recently, Kavanaugh et al. [23] proved as well that segmented polyurethanes prepared with β-glycerol phosphate as a biologically active chain extender, supported human mesenchymal stem cell adhesion, growth, and osteogenic differentiation. The PU system studied by Kavanaugh et al. was synthesized by using poly(ε-caprolactone)diol, 4,4′-methylene bis(cyclohexyl isocyanate), and biologically active compounds such as ascorbic acid, l-glutamine, β-glycerol phosphate, and dexamethasone as chain extenders [23]. In brief, the use of β-glycerol phosphate as a chain extender has improved the biological activity of polyurethane applicable as a material for bone regeneration. Furthermore, glycerophosphates have high potential for mineralization, proper adhesion and proliferation and therefore the attempted GPCa chain PU contrasts with the approach by Gornal et al. [16] where it was used as a filler.

In this paper we describe the synthesis and characterization of the PEEURs designed for bone tissue regeneration. PEEURs were synthesized by using 1,6-hexamethylene diisocyanate (HDI), poly(ethylene glycol) (PEG), α,ω-dihydroxy(ethylene-butylene adipate) (Polios), 1,4-butanediol (BDO) as a chain extender and GPCa as a modifier used to stimulate bone tissue regeneration. The obtained unmodified (PUR) and GPCa modified (PURs-M) PEEURs were studied with various techniques in order to confirm the reactivity of GPCa with the urethane prepolymer, to study its chemical composition, surface morphology, hydrophilic character, mechanical properties, in vitro biocompatibility and short-term interactions with selected acidic, basic and oxidative environment. Moreover the calcification study was performed to establish if the GPCa modifier improved the bioactive character of the obtained PUR-M materials. Furthermore, with the use of selected samples, porous scaffolds were obtained by using the SC/PL technique combined with TIPS. According to performed studies the obtained PURs-M may be a suitable candidate for bone tissue engineering and further studies are being developed by our team in this field.

## 2. Experimental

### 2.1. Poly(ester ether urethane)s Synthesis

PEEURs were synthesized by the standard two step polymerization procedure with urethane prepolymer intermediate [1,6]. The urethane prepolymer was obtained in the reaction of polyester α,ω-dihydroxy(ethylene-butylene adipate) (PEBA, trade name Polios 55/20; Purinova, Bydgoszcz, Poland) (63 wt %),poly(ethylene glycol) (PEG) (14 wt %) and aliphatic 1,6-hexamethylene diisocyanate (HDI) (Sigma Aldrich, Poznań, Poland) (23 wt % ). In the second step the chain extender—1,4-butanediol (BDO) (POCH, Gliwice, Poland)—Was added to the urethane prepolymer to obtain PEEUs with a molar ratio of free isocyanate groups (NCO) (in the urethane prepolymer) to hydroxyl groups (OH) of the chain extender BDO equal to NCO/OH = 0.9:1.

**Figure 1:**
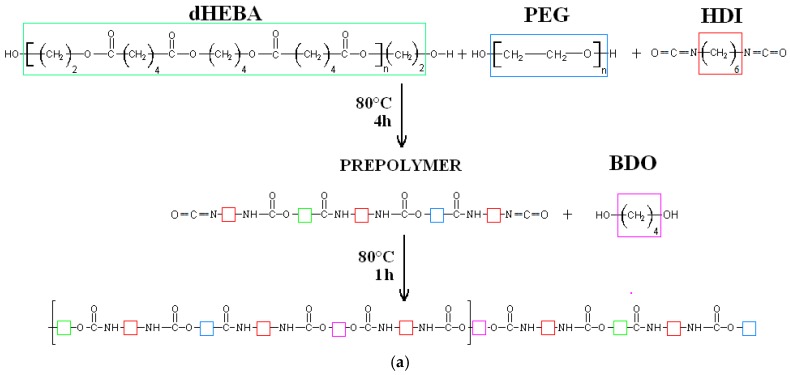

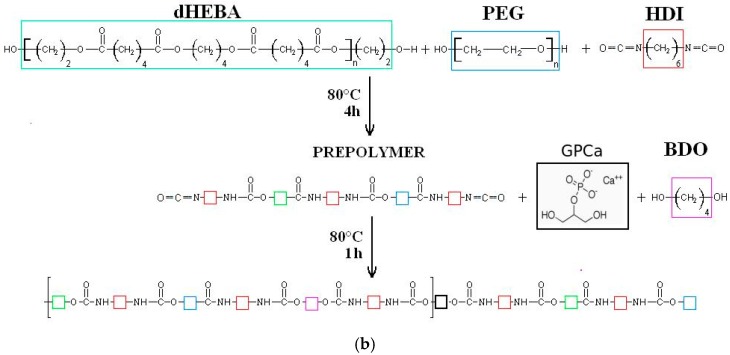
Synthesis of unmodified (**a**) and calcium glycerolphosphate (GPCa)-modified (**b**) poly(ester ether urethane)s (PEEURs).

The synthesis of modified PEEURs (PUR-M) was as follows: In the first step the urethane prepolymer was obtained in the reaction between PEBA, PEG, and HDI. In the second step the 10 wt % of GPCa (Sigma Aldrich, Poznań, Poland) calculated per mass of the prepolymer was added at 80 °C and stirred for 4 h (the mass ratio of urethane prepolymer to GPCa was equal to 1:0.25). In the next step the chain extender BDO was added to obtain PUR-M with a molar ratio of free isocyanate groups (NCO) (in the urethane prepolymer) to hydroxyl groups (OH) of chain extender BDO equal to NCO/OH = 0.9:1. Reaction of unmodified and modified polyurethanes is presented in Figure 1.

### 2.2. Characterization Methods

#### 2.2.1. Indications of Free Isocyanate Groups (F_NCO_) by the Acidimetric Method

Indication of free isocyanate groups is a standard procedure in the case of PURs obtained by a two-step polymerization method. Its aim is to establish the time, after which the requiredamount of unreacted diisocyanate groups (NCO) in the prepolymerization reaction takes place. The determination of free isocyanate groups (F_NCO_, %) was performed according to the PN-EN 1242:2006 standard.

#### 2.2.2. Fourier Transform Infrared Spectroscopy (FTIR)

The FTIR of the solid PUR and PUR-M was performed by an FTIR Nicolet 8700 Spectrometer (Thermo Fisher Scientific, Waltham, MA, USA) with Specac’s Golden Gate module and single reflection diamond ATR unit to determine the influence of the processing technique on the composition of the obtained materials. The studied spectral range was from 4000 to 500 cm^−1^ averaging 254 scans per sample with a resolution of 4 cm^−1^.

#### 2.2.3. Raman Spectroscopy

Raman spectra were performed by using Monovista CRS + spectrometer provided by S&I Ltd., (Warstein, Germany) which was operated by VistaControl 4.1 software (S&I Ltd., (Warstein, Germany). Data about the polymers’ structure was obtained by using a 532 nm green laser with the scanning power reduced to 10 mV. The best suitable grating was set to 1800 grooves per mm and was chosen to provide resolution of about 0.5 cm^−1^. The slit was set to standard 100 micrometers and the objective used to scan in Point by Point, 2D mode was a long focal-length Olympus with 100 magnification rate. All spectra were obtained by gaining two accumulations within 10 s time frame. Each scanning point from which the Raman spectra were taken had approximately 1 μm^2^ surface.

#### 2.2.4. Scanning Electron Microscopy with Energy Dispersive X-ray Spectroscopy (SEM/EDX)

SEM was performed with the use of a Zeiss Scanning Electron Microscope EVO-40 (Jena, Germany) (different magnifications were used: 3000×, 1000×, and 250×). The SEM instrument was integrated with an energy dispersive X-ray (EDX) microanalyzer (Jena, Germany) for elemental analysis. Prior to the study PURs were covered with a conductive layer of gold by sputter coater Quorum 150T E. To study the morphology of the obtained PURs, PURs-M and the scaffolds ImageJ^®^ software (U.S. National Institutes of Health, Bethesda, MA, USA) was used.

#### 2.2.5. Static Contact Angle Determination

The contact angle as well as the surface free energy of the materials’ surfaces weredeterminedat room temperature by using a Kruss Goniometer G10 (KRÜSS GmbH, Hamburg, Germany) with drop shape analysis software. The water contact angle of the 10 samples was evaluated by static contact angle measurements using the sessile drop method. Surface free energy was calculated by using measurements performed according to the Acid-Base method (AB) [24] by static contact angle studied with the use of three liquids: Water, ethylene glycol, and formamide.

#### 2.2.6. Mechanical Properties

Tensile strength (*T*_SB_) and elongation at break (ε_b_) were studied using the universal testing machine Zwick & Roell Z020 (Zwick Roell Polska Sp. z o.o. Sp.K., Wrocław, Poland) according to PN-EN ISO 527-2:2012 with a crosshead sped of 500 mm/min. Samples of sixwere used for this study.

Hardness was measured by using the Shore method according to PN-EN ISO 868:2004. Obtained data were presented with Shore degree (°Sh D and °Sh A). Samples of 10 were used for this study.

#### 2.2.7. Short-Term Interactions Study Performed in Selected Environments

PURs were cut into round samples of 0.5 cm^2^ area. Prepared samples were dried and weighed in a thermobalance (Radwag, Radom, Poland) (RADWAG MAX50/SX) set at 60 °C. Then, 6 samples of each studied PUR materials were placed in a 24-well cell culture plate filled with selected media: Oxidative solution of 0.1 M CoCl_2_/20% H_2_O_2_; acidic solution of 2 N HCl or basic solution of 5 M NaOH. Samples were incubated in the selected media at 37 °C. The mass change of the samples was examined after 15 days for oxidative, acidic, and basic media. Samples mass change measurement was as follows: Samples were taken out from the container and put into a paper sheet to reduce the medium excess. Then, samples were placed in the thermobalance (set at 60 °C) where they were weighed to constant mass. The results are the arithmetic mean of six measurements.

The changes at the PUR and PUR-M surface were monitored by optical microscopy (OM) performed with the use of a Bresser microscope(Bresser GmbH, Rhede, Germany) at a magnification of 20×.

#### 2.2.8. In Vitro Cytocompatibility

The cytotoxicity assay was performed by using selected PUR and PUR-M samples. To examine the cytotoxicity of the obtained materials, the extract was prepared and tested with the use of a C2C12 cell line according to ISO 10993-5:2009 standard. In order to obtain extract the sterile PUR or PUR-M samples were incubated at the ratio 1:100 (*w*/*v*) in cell culture medium (Dulbecco’s Modified Eagle’s Medium, DMEM) (Gibco, Gaithersburg, MD, USA) supplemented with 10% fetal bovine serum (FBS) (Gibco), l-glutamine (1% solution in medium) (Gibco), 1% antibiotic–antimycotic mixture for 24 h at 37 °C under continuous steering.

##### Cell Viability Assay

MTT (3-(4,5-dimethylthiazol-2-yl)-2,5-diphenyltetrazolium bromide) assay was used for assessing cell metabolic activity. C2C12 cells were split into 24-well plates at 30,000 cells per well and grown for 24 h in 500 μL of culture medium. Cells were incubated for 72 h with the matrices’ extracts. Then, the MTT assay of viable cells was used in accordance with the manufacturer’s recommendations (Sigma Aldrich, St. Louis, MO, USA). The reaction product was quantitatively determined by an Absorbance Reader BioTek EL*800 (BioTek Instruments, Inc., Winooski, VT, USA) at a wavelength of 570 nm. The viability of the untreated cells was counted as 100.

#### 2.2.9. Calcification Study

Golomb and Wagner’s Compound was used to perform the calcification study. The calcification metastable solution consisted of 3.87 millimole (mM) CaCl_2_, 2.32 mM K_2_HPO_4_, yielding a ratio of calcium to phosphate (Ca/PO_4_) = 1.67, and 0.05 M Tris Buffer (in this study C_4_H_11_NO_3_) dissolved in one mL of reverse osmosis (RO) water [25]. PUR and PUR-M samples were cut into round samples of 0.5 cm^2^ area. Prepared samples were dried and weighed in a thermobalance (RADWAG MAX50/SX) set at 60 °C. Then, 6 samples of each studied PUR materials were placed in a 24-well cell culture plate filled with Golomb and Wagner’s Compound. The progress of the calcification was studied by SEM and EDX after 21 days.

#### 2.2.10. Scaffold Fabrication

PUR or PUR-M was dissolved in 1,4-dioxane (POCH, Gliwice, Poland) at a concentration of 20% *w*/*v*. Then, sodium chloride, of crystal size in the range of 0.6–0.4 μm, was added to the polyurethane solution until complete saturation of the solution occurred. Formulated PUR (or PUR-M)-salt saturated solution was transferred into the stainless steel mold of the size 2.5 cm × 2.5 cm × 2.5 cm and placed at −20 °C for 24 h to direct the solvent crystallization and to fabricate scaffolds of local anisotropy where the porosity of the scaffolds was of controlled pore size and porosity [26,27,28,29]. Then scaffolds were removed from the mold and immersed in warm (40–50 °C) bidistilled water, where for 7 days the sodium chloride crystals were washed out. The water was changed twice a day. Finally, the samples were dried at 60 °C for 24 h.

## 3. Results and Discussion

The impact of the prepolymer modification time on the decrease of the free isocyanate groups in it is outlined below.

Table 1 presents the influence of the prepolymer modification time on the decrease of the free isocyanate groups present in it.

Table 1 shows that the prepolymerization reaction takes place in a similar way for both PUR and PUR-M materials until the 4th hour of the synthesis, when the GPCa modifier was added to the system. Incorporation of the GPCa modifier into the system caused a slightly higher F_NCO_ decrease for PUR-M in comparison to PUR after 1 h of modification (8.54 ± 0.02% and 8.43 ± 0.03% respectively). After 5 h of prepolymer modification with GPCa the F_NCO_ reached the previously established 8 wt % and the reaction was recognized as finished. It can be concluded that the NCO groups of diisocyanate react with OH groups present in the GPCa modifier at the adjusted modification conditions. It suggests that GPCa was incorporated into the PUR structure through covalent bonds.

### 3.1. Fourier Transform Infrared Spectroscopy (FTIR)

**Figure 2:**
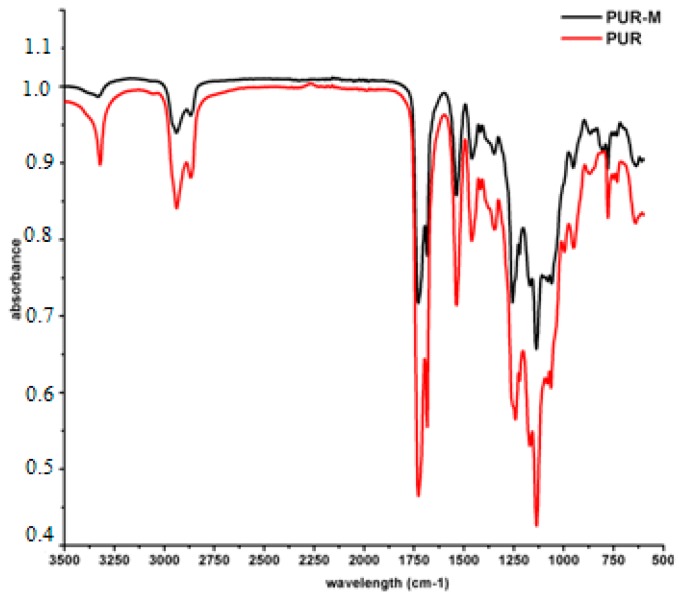
FTIR spectra of PUR (black) and PUR-M (red).

Figure 2 presents the FTIR spectra of PUR and PUR-M.

Analysis of FTIR spectra (Figure 2) revealed the presence of functional groups characteristic for PURs composition; i.e., urethane linkages (see Table 2). Thus, conditions designed to carry out PURs synthesis were suitable and provided PUR product. The expanded base of the NH stretching band (3392 and 3326 cm^−1^) for PURs suggested the presence of “free” and hydrogen bonded HS in the obtained material respectively [30,31]. In the case of PUR-M the expanded base of the NH stretching band was observed as well, but its intensity relating to the “free” NH (3389 cm^−1^) in the PUR-M composition was decreased. Conversely to the PURs the PUR-M revealed the well-shaped NH stretching band related to the moderate and strong hydrogen bonds present in the HS of PUR chains [32,33]. The observed C=O stretching band confirmed the presence of ester and urethane linkages, which were “free” (1728 cm^−1^) and hydrogen bonded (1680 cm^−1^) for both PUR and PUR-M [32,33]. It can be pointed out here that the PUR-M appears to have a large number of hydrogen bonded urethane linkages in comparison to PURs. Performed FTIR analysis confirmed the presence of urethane linkages. Furthermore, the FTIR spectra of PUR-M showed that GPCa modification represents a higher level of hydrogen bonds between HS in PUR chains. Some differences in band intensities (1462–945 cm^−1^) between PURs and PURs-M were observed which might be related as well to the presence of GPCa molecules in the PUR system [34].

### 3.2. Raman Spectroscopy

**Figure 3:**
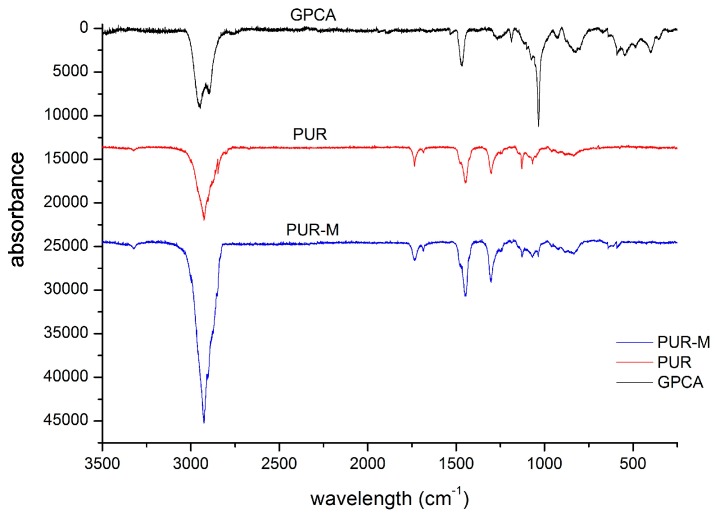
Raman spectra of PUR and PUR-M.

In Figure 3 the Raman spectra of obtained PUR and PUR-M are presented, of which the band assignments are given in Table 3 and Table 4.

The performed Raman spectroscopy results were complimentary to the FTIR study (Table 4 and Table 5). Thus, it confirmed the presence of strongly hydrogen bonded urethane groups in the PUR-M structure as well as introduction of the GPCa modifier into the main chain of PUR at least at one-side.

### 3.3. Scanning Electron Microscopy with Energy Dispersive X-ray Spectroscopy(SEM/EDX)

**Figure 4:**
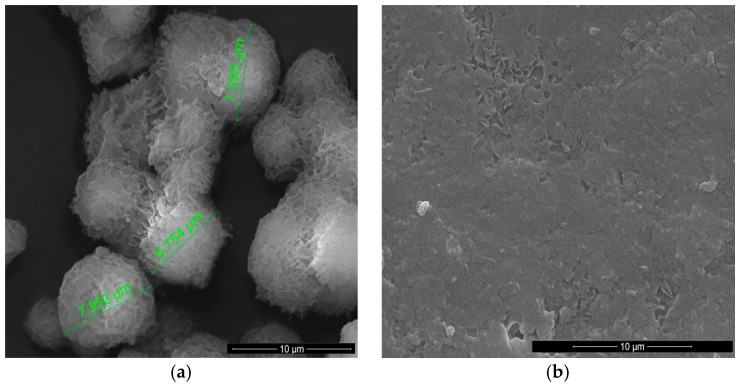

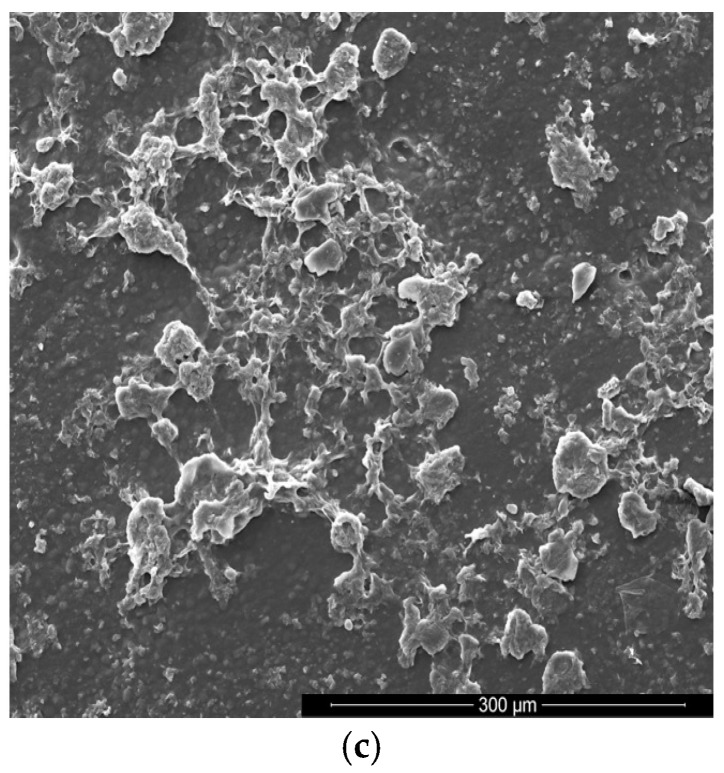
SEM images of the GPCa modifier (**a**) and the surface of PUR (**b**) and PUR-M (**c**).

Figure 4 shows the SEM images of the GPCa modifier and the obtained PUR and PUR-M surface.

Figure 4a shows an image of GPCa used as a modifier. The particles resemble a spherical shape and their sizes are in the range of 6.754–7.952 μm. Figure 4b shows the image of the PURs surface, which is rather homogeneous and without visible inclusions. In Figure 4c it can be seen that the surface of PUR-M modified with GPCa is homogenous over 80% with little inclusions visible at the surface top. These inclusions can be related to the GPCa, which did not react with prepolymer and was partially enclosed in the polyurethane matrix. It suggests that a large amount of used GPCa modifier was incorporated into the PUR structure by covalent bonding, which is consistent with FTIR and Raman spectroscopy.

### 3.4. Contact Angle

In Table 5 are gathered the values of the contact angles and total surface free energy studied for the obtained PUR and PUR-M.

The obtained PURs and PURs-M had water contact angles in the range from 72° (PUR) to 57° (PU-M) (Table 5). The contact angles studied in formamide and ethylene glycol were slightly lower in comparison to the values of the water contact angle. Addition of GPCa caused a decrease of the contact angle independently of the solvent used in the study. The total surface free energy is higher for PURs-M (59 mN/m) than for PURs (32 mN/m). This trend was observed independently of the method used for its examination (Table 5).

### 3.5. Mechanical Properties

**Figure 5:**
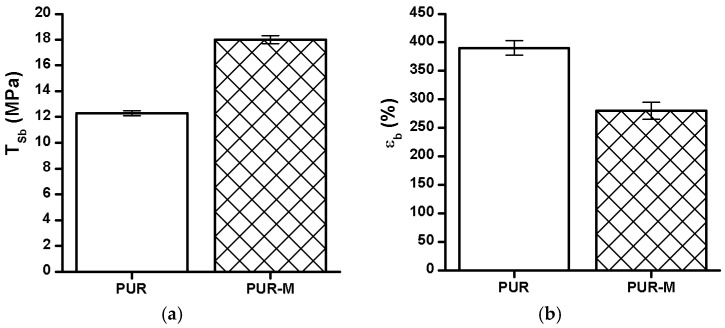

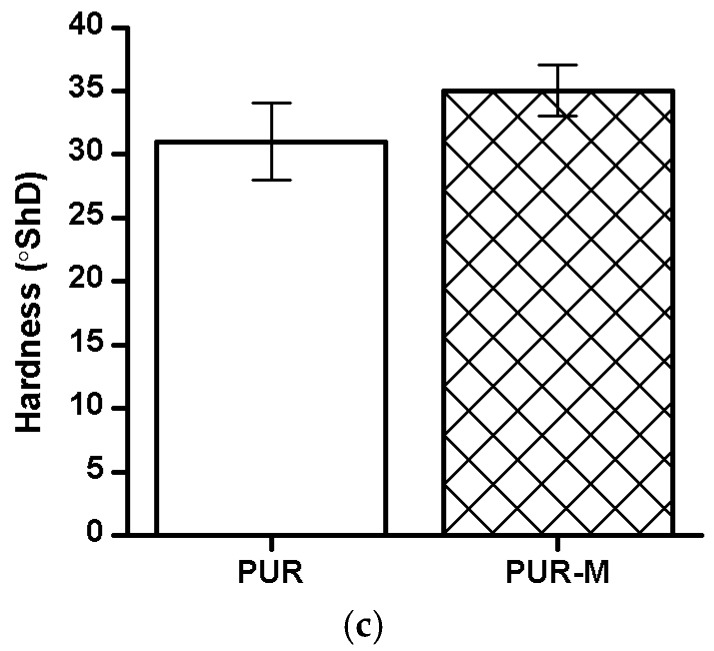
The *T*_Sb_ (**a**), ε_b_ (**b**), and hardness (**c**) of obtained PUR and PUR-M.

The *T*_Sb_, ε_b_ and hardness of the obtained PUR and PUR-M are shown in the Figure 5.

The *T*_Sb_ of PURs-M (18 MPa) was 6 MPa higher than for PURs (12 MPa). In the case of ε_b_ the introduction of GPCa modifier caused a decrease of this property from 390 ± 13% (PURs) to 280 ± 15% (PURs-M). The hardness was comparable for both PURs and PURs-M (31 ± 3 °ShD for PURs and 35 ± 2 °ShD for PURs-M).

### 3.6. In Vitro Cytocompatibility

**Figure 6:**
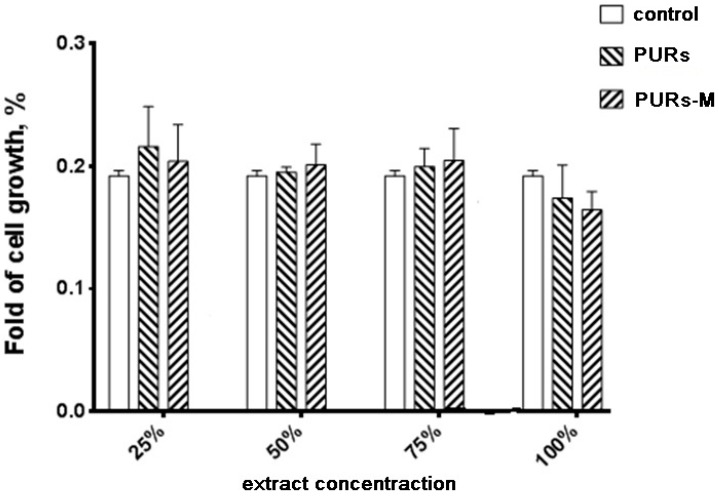
The effect of PUR and PUR-M extracts on the in vitro growth of C2C12 cells studied by MTT assay after 72 h (* *p* < 0.05).

Figure 6 shows the MTT assay results performed by using PURs and PURs-M extracts at different concentrations.

Performed in vitro cell studies revealed good biocompatibility of the obtained PUR and PUR-M materials independent of the extract concentration. In the case of extract concentrations in the range of 25–75% as light improvement of cell growth was noted for PURs-M in comparison to the controls. Only in the case of undiluted extracts (100%) was the cell viability of PURs and PURs-M slightly lower in comparison to the controls, but still in the range of good biocompatibility.

### 3.7. Short-Term Interactions Study Performed in Selected Environments

Table 6 shows the mass loss of PURs and PURs-M noted after the short-term interactions study (15 days) performed with the selected media of acidic, basic, and oxidative environment.

Indicated mass loss was noted for both PUR and PUR-M materials (Table 6). Thus, both types of obtained materials may be considered as possibly degradable. In a strongly basic environment both materials had similar values of mass loss, which was over 50%. The GPCa modification (PURs-M) caused the increase of degradation rate of about 4% in comparison to PURs. In the case of acidic environment the mass loss was over 30% for both PURs and PURs-M, and this mass loss was higher by 3% for PURs than for PURs-M. In the oxidative environment PURs-M were slightly more stable than PURs, but the mass loss did not exceed 5%.

**Figure 7:**
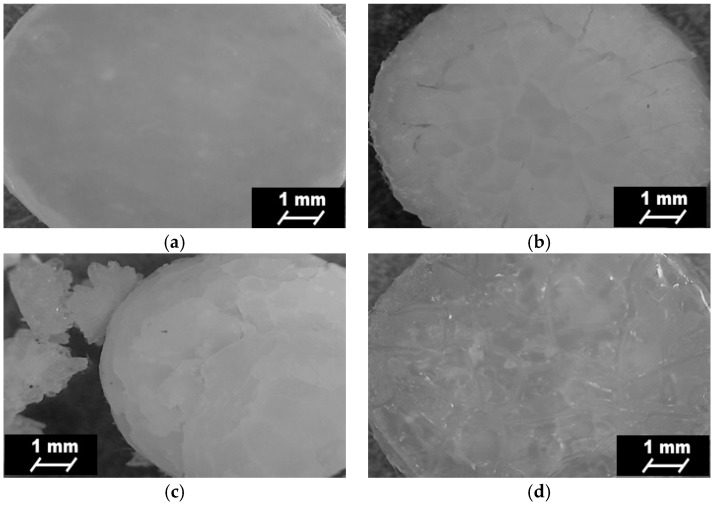
Optical microscope images of PURs before (**a**) and after 15 days of short-term interactions study performed in the selected environments: basic (**b**); acidic (**c**); and oxidative (**d**).

**Figure 8:**
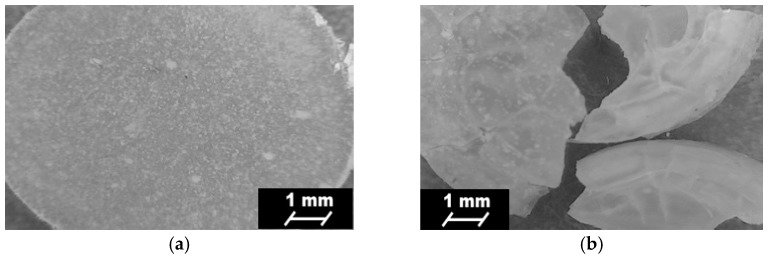

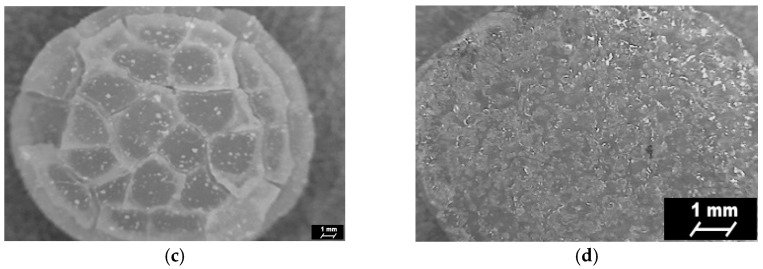
Optical microscope images of PURs-M after (**a**) and after 15 days of short-term interactions study performed in the selected environments: basic (**b**); acidic (**c**); and oxidative (**d**).

The surface changes of PURs and PURs-M were monitored before and after the 15 days of short-term interactions study. Figure 7 and Figure 8 show the surface changes at the time of the performed study.

Optical microscopy showed the visible changes, which took place on the surface top of the obtained PURs and PURs-M after 15 days of the short-term interactions study performed for different environments (Figure 8). It can be observed that PUR-M materials are more sensitive in the basic and acidic environment due to the fact that defragmentation of these materials occurs. The indicated materials’ defragmentation, according to the references, may concern an initial step of polymer degradation [32,42]. The optical microscopy images confirmed that both PURs and PURs-M are resistant to the oxidative environment.

### 3.8. Calcification Study

**Figure 9:**
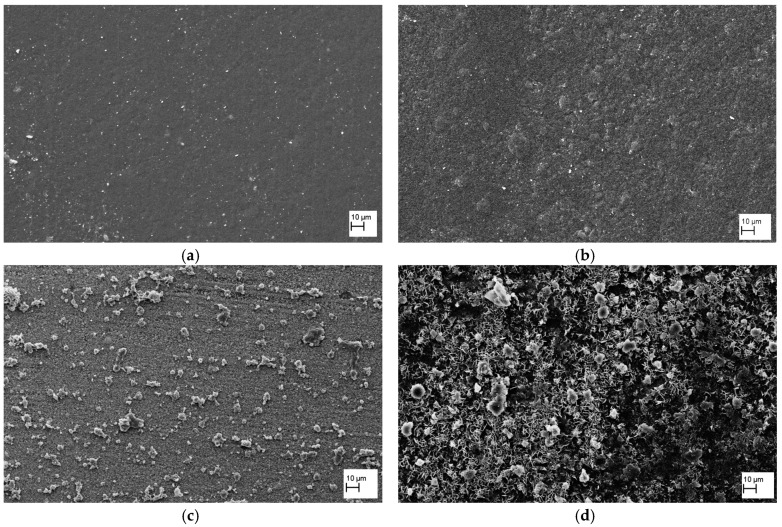
SEM images of PUR (**a**) and PUR-M (**b**) before calcification study and images of PUR (**c**) and PUR-M (**d**) after calcification study.

SEM images of PURs and PURs-M before and after the calcification study are presented in Figure 9.

**Figure 10:**
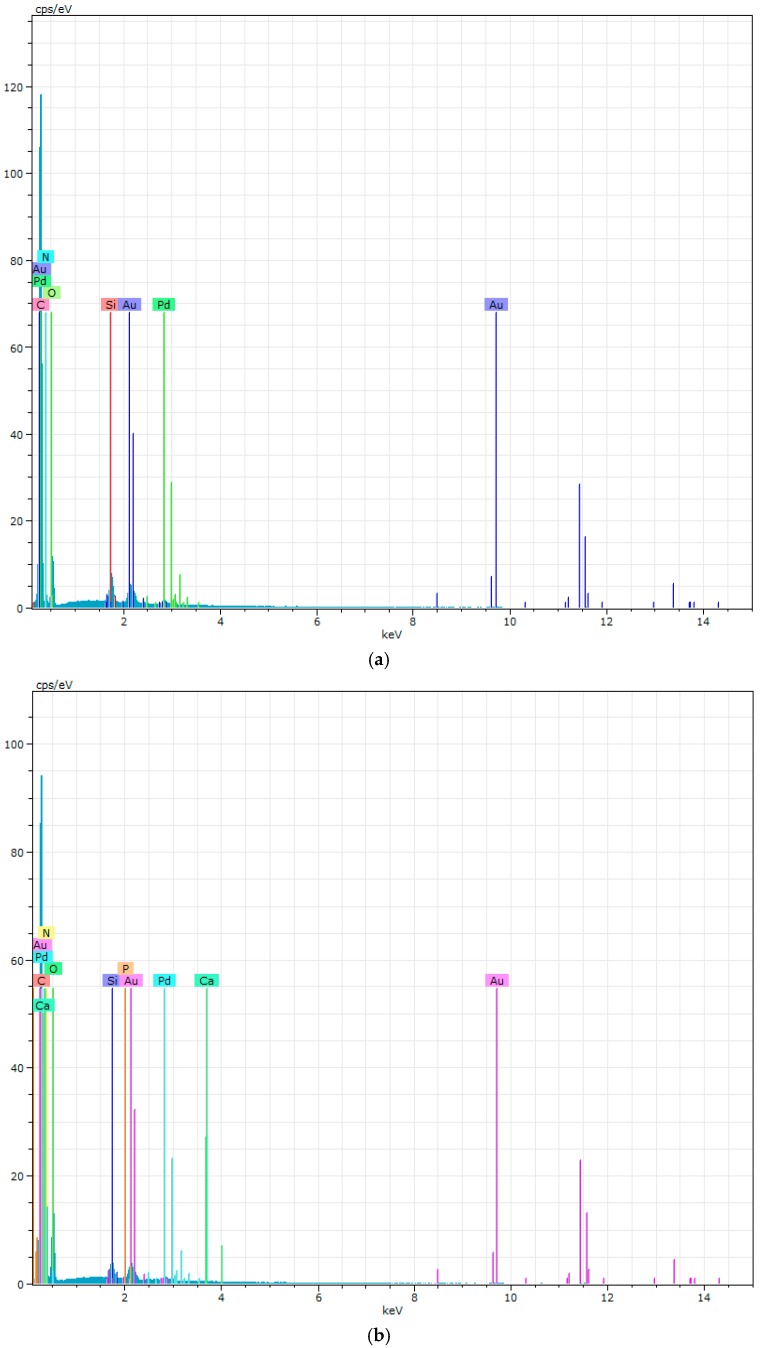
The EDX of PURs (**a**) and PURs-M (**b**) before calcification study.

**Figure 11:**
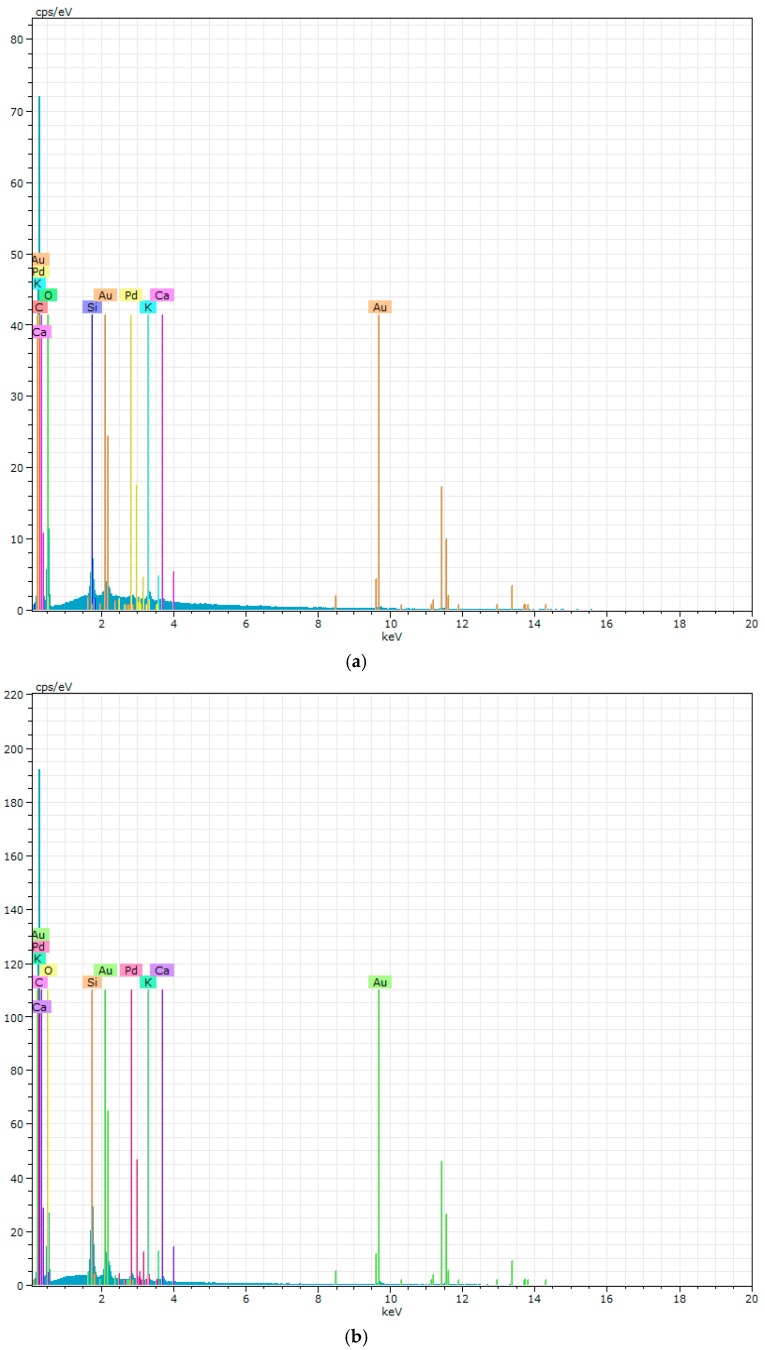
The EDX of PURs (**a**) and PURs-M (**b**) after calcification study.

As can be noted in Figure 9 the PURs do not have an excessive ability to calcification. This is in contrast to the PURs-M, on whose surface a significant deposition of calcium was indicated. The confirmation of the calcification progress on the obtained materials surface was achieved due to the EDX study, which is presented in Figure 10 and Figure 11.

Figure 10 showes that PURs do not contain calcium at their surface prior to the calcification study. In the case of PURs-M the calcium content is approximately 50% which is related to the presence of GPCa modifier. After the calcification study (Figure 11) the calcium amount at the materials’ surfaces increase by over 50% for PURs and 110% on PURs-M. Thus, the GPCa modification has a significant impact on the calcification progress.

### 3.9. Fabrication of PUR and PURs-M Scaffolds

**Figure 12:**
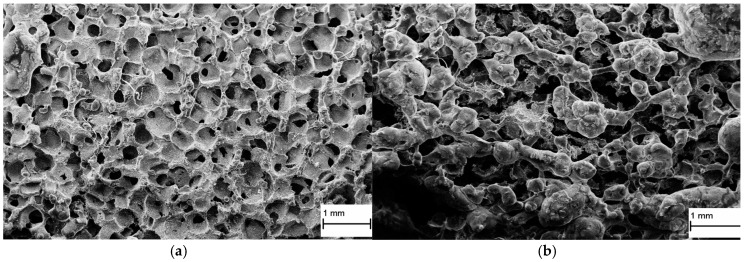
The PUR (**a**) and PUR-M (**b**) scaffold fabricated by SC/PL in combination with TIPS.

Obtained PURs and PURs-M met the requirements of biomaterials used for bone tissue engineering. Their mechanical, physicochemical biological characteristic was suitable for bone tissue engineering. Thus, in the next step we made an attempt to fabricate, with the use of PURs and PURs-M, the porous scaffold by using SC/PL combined with TIPS. Figure 12 shows the SEM micrographs of the obtained porous scaffolds.

Obtained PUR had pore sizes in the range of 36–100 μm and a porosity of approximately 77%. On the other hand, PUR-M scaffolds had pore size in the range of 50–100 μm and a porosity of approximately 82%. This morphological characteristic is suitable, according to the references [8], for using such scaffolds in bone tissue engineering applications. Furthermore, in the case of PURs-M scaffolds, particles of the GCPa modifier were visible. Thus, they were not washed out of the scaffold during the scaffold fabrication process and according to the biocompatibility and calcification study, this may be a useful compound stimulating the regeneration of native bone tissue [43]

**Figure 13:**
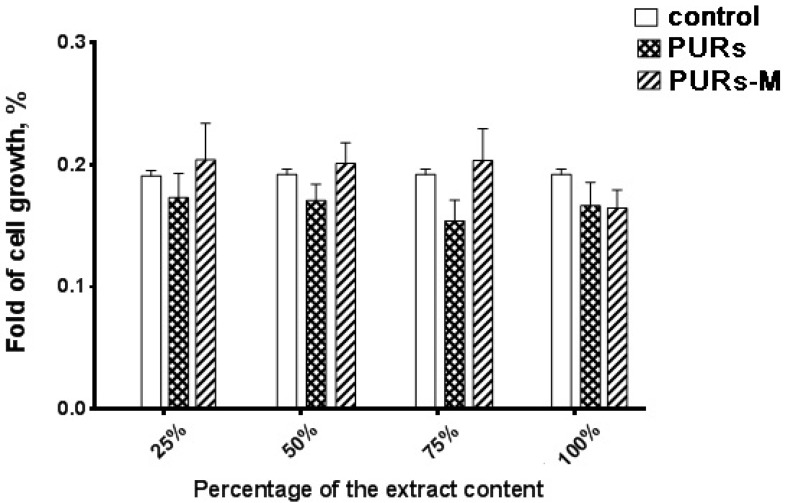
The effect of PUR and PUR-M scaffolds’ extracts on the in vitro growth of C2C12 cells studied by MTT assay after 72 h (* *p* < 0.05).

According to the proper morphology of the obtained porous scaffolds their biocompatibility was studied as well. Figure 13 shows the cells viability after 72 h of MTT assay.

The main conclusion coming from the analysis of Figure 13 is the fact that the obtained PURs-M possesses better biocompatibility than PURs. Thus, it confirms the beneficial effects of the employed GPCa modifier. The proliferation of cells of the PUR-M extracts was observed at concentrations between 25–75%. PURs had lower cells viability in comparison to the PURs-M. In the case of undiluted extracts (100%) the cells viability was comparable for both PUR and PUR-M scaffolds.

## 4. Discussion

Bone tissue engineering is a demanding field of strictly described requirements of biomaterials, which may be used for bone tissue scaffold fabrication. Accordingly of the many biomaterials used in this field PU seems to be the most suitable candidate. This is due to its ease of modification to attain a bioactive material as well as its suitable mechanical property design related to the raw materials selection for its synthesis [14,44,45,46,47].

In this paper we described the synthesis of PEEURs carried out with the use of selected raw materials such as aliphatic HDI, polyester (Polios,) and polyether (PEG) polyols, with BDO chain extender to reach the requirements of biocompatible biomaterials for medical applications. The GPCa modifier was selected according to the literature, which describes it as a compound that can improve the bioactivity of the material as well as stimulating bone tissue regeneration. The successful synthesis of PURs was confirmed by FTIR and Raman spectroscopy, which revealed the formation of urethane bonds. Application of GPCa modifier improved hydrogen-bond formation in the PURs-M structure compared to the PURs (see FTIR analysis). Spectroscopic studies and F_NCO_ determination confirmed the fact that GPCa is partially covalently bonded with the PUR chain. This is possible due to the hydroxyl groups present in the GPCa chemical structure. The SEM image of the PUR-M surface was in good agreement with the FTIR analysis due to the fact that it revealed the presence of a homogenous surface (about 80%) of this material, with only little inclusions visible at the top. The presence of these inclusions could be related to the GPCa, which did not react with prepolymer and was partially enclosed in the polyurethane matrix in the form of the filler. This filling effect of GPCa occurring was beneficial in the case of the biocompatibility and calcification study and may be useful as bioactive stimulant to improve bone tissue regeneration. The contact angle was decreased for the PURs-M (57°) in comparison to the PURs (72°). Thus, the addition of GPCa improved in a superior way the hydrophilic characteristic of the obtained materials, which may be beneficial for cell growth. According to Guelcher et al. the contact angle of the polymer surface in the range of 45–76° supports the attachment of mammalian cells, which can have a beneficial influence on the absorption of albumin and a preservative coupler of live tissues [48]. The total surface free energy (59 mN/m for PURs-M and 32 mN/m for PURs respectively) was in good agreement with the contact angle study and is suitable for cell growth only for PURs-M according to the references [11,49,50]. In further studies the total surface free energy can be increased by increased GPCa addition into the materials. Most researchers have come to the conclusion that rather a surface charge (either positive, or negative) is the most important, not the surface free energy [49]. According to this, a modification of a hydrophilic but not-charged polyurethane surface with bipolar calcium β-glycerophosphate is a good option for the optimization of cellular adherence at the PU surface. The mechanical properties of the obtained PURs (*T*_Sb_ = 12 MPa, ε_b_ = 390 ± 13%) and PURs-M (*T*_Sb_ = 18 MPa, ε_b_ = 280 ± 15%) were more suitable for bone tissue engineering in the case of PUR-M. The materials used in bone regeneration must have a *T*_Sb_ in the range of 1.5–38 MPa for human cancellous bone regeneration or 35–283 MPa for human cortical bone regeneration [51]. Thus, the obtained PURs and PURs-M may find an application as biomaterials for scaffold fabrication of human cancellous bone. The hardness was comparable for both PURs and PURs-M (31 ± 3 °ShD for PURs and 35 ± 2 °ShD for PURs-M). The short term interactions study showed that both PURs and PURs-M are sensitive to basic and acidic environment, while in an oxidative one they remain stable. Thus, this is consistent with references [52]. The observed superior mass decrease, in the case of basic (over 50%) and acidic (over 30%) environment, after 15 days of the short-term interactions study may be a sign of the first step of material degradation called defragmentation [53]. This was confirmed by the optical microscopy studies, which showed defragmentation of the obtained PURs and more favorably PURs-M. The SEM and EDX verification of the calcification study showed significant improvement in the progress of calcification, the process needed in the case of bone regeneration [11,16,35,54], for PURs-M. Thus, the GPCa modification is a superior factor, which improves calcification, which was indicated by the peaks of Ca present in the EDX spectra. After the calcification study the calcium amount at the materials’ surfaces increased over 50% for PURs and 110% on PURs-M. According to the satisfactory physicochemical, mechanical, and biological characteristics of the obtained PURs and PURs-M the fabrication of the scaffolds was performed by using the SC/PL technique combined with TIPS. Obtained PUR scaffolds had pore sizes in the range of 36–100 μm and a porosity of approximately 77%. On the other hand, PUR-M scaffolds had a pore size in the range of 50–100 μm and a porosity of approximately 82%. This morphological characteristic is suitable, according to the references, for using such scaffolds in bone tissue engineering applications. Forthermore, in the case of PURs-M scaffolds particles of GCPa modifier were visible, which were not washed out during the scaffold fabrication process. Thus, according to the results of the MTT assay, it can be concluded that PURs-M may be a suitable candidate for bone tisue engineering applications [43].

## 5. Conclusions

In this paper we reported the synthesis and characteristic of PEEURs, obtained by using raw materials (HDI, Polios, PEG, and BDO), selected to reach high biocompatibility of the materials. Moreover, we successfully modified the PEEUR chains by incorporating into them GPCa modifier, confirmed by various techniques. The stronger hydrogen bonding, lower contact angle, and higher total surface free energy as well as the more suitable mechanical properties and good biocompatibility of PURs-M let to the conclusion that these materials possess satisfactory characteristics of materials dedicated to bone tissue engineering. Further studies of calcification of these materials indicated the superior effect of GPCa on these processes. Moreover, the short term interactions study revealed that the obtained PUR-M materials undergo gradual degradation in selected basic and acidic environment by chain defragmentation and such degradable materials are being widely developed for tissue scaffolds. The obtained porous scaffolds, by using the SC/PL technique combined with TIPS, represented suitable pore sizes (36–100 μm) and porosity (77–82%) to serve as templates for bone tissue regeneration. Moreover, the biocompatibility of PURs-M scaffolds was superior in comparison to PURs. Thus, further studies in this direction will be developed in our team.

## Figures and Tables

**Table 1:** The impact of the prepolymer modification time on the decrease of free isocyanate groups present in it.

Time of prepolymeryzation (h)	Content of the free isocyanate groups in the unmodified prepolymer (NCO )	Content of the free isocyanate groups in the modified prepolymer (NCO )
	PEEUR	PEEUR-M
0	10.14 ± 0.03	10.14 ± 0.03
1	9.04 ± 0.01	9.04 ± 0.01
2	8.83 ± 0.03	8.83 ± 0.03
3	8.54 ± 0.03	8.54 ± 0.03
4	8.54 ± 0.02	8.54 ± 0.02
5	8.54 ± 0.02	8.43 ± 0.01 *
6	8.53 ± 0.03	8.33 ± 0.03
7	8.53 ± 0.01	8.14 ± 0.03
8	8.53 ± 0.02	8.12 ± 0.02
9	8.53 ± 0.01	8.12 ± 0.03

* addition of the GPCa modifier.

**Table 2:** Spectral data and band assignments of FTIR analysis presented in Figure 2.

PUR	PUR-M	Band	Description
Wavelength (cm^−1^)			
3392 3326w	3380 3318w	νNH	stretching of NH groups, hydrogen bonded withthe C=O of the ester group present in macrodiol and in GCPa modifier
2942m 2868m	2942m 2868m	νCH_2_	stretching of aliphatic asymmetric and symmetric CH_2_ groups present in the PUR chain and in GCPa modifier
1728m	1728s	νC=O	stretching of C=O in the ester and urethane groups, which were not bonded
1680m	1680s	νC=O	stretching of C=O groups which formed hydrogen bonds
1535m	1535s	νC–N	stretching between CN in urethane group
1462w 1416w 1350w	1459m 1415m 1347m	δCH_2_	deformation vibrations of aliphatic CH_2_ groups present in the PUR and GPCa modifier: bending, wagging, scissoring in plane
1260w 1214m	1238s 1216s	νC–(C=O)–O	stretching vibrations of –C–(C=O)–O– of ester group, not hydrogen bonded
1170m	1170s	νNH–(C=O)–O	stretching vibrations of –NH–(C=O)–O– of urethane group
1135m 1080m 1058m 947w	1134s 1061s 993s 949s	νC–(C=O)–O νC–O	stretching vibration of hydrogen bonded –C–(C=O)–O–,
868w 813w 775w 731w 636w	867s 777s 730s 638s	δCH_2_, δNH δOH	out of the plane deformation of CH_2_ and CH_3_ groups as well as NH and OH groups

**Table 3:** Raman spectra band assignments for obtained PURs.

Wavelength (cm^−1^)	Assignments *
3328	stretching vibrations of N–H in urethane groups (as for II-ary amides)
2925, 2890	the strongest polarized stretching vibrations of asymmetric and symmetric CH_2_ groups present in PUR chains.
1735, 1685	stretching vibrations of carbonyl groups present in macrodiol Polios 55/20 and urethane groups (as for II-ary amides) respectively
1480, 1450, 1443, 1424	strong planar deforming vibrations (scissoring) of CH_2_ groups
1302	swinging and bending vibrations of CH_2_ groups outside of plane
1249	stretching vibrations of C–N in urethane groups
1127, 1068	stretching asymmetric and symmetric vibrations of C–O–C in ester groups respectively
1035–1095	stretching vibrations of saturated aliphatic chains C–C–C–C
962, 935, 885, 832	swaying vibrations of CH_2_ groups in different positions and deforming bending vibrations outside of the plane of N–H groups
610	deforming vibrations outside of the plane of ester groups and their fluctuations

* [35,36,37,38].

**Table 4:** Raman spectra band assignments for GPCa modified PURs.

Wavelength (cm^−1^)	Assignments *
3315, 3326	stretching vibrations of N–H in urethane groups (as for II amides), P–O–Ca stretch
2925, 2883	the strongest polarized stretching vibrations of asymmetric and symmetric CH_2_ groups present in PUR chains. Analogic CH_2_ in H_2_C–O–P–O stretch phonons included
1733	stretching vibrations of carbonyl groups present in macrodiol Polios 55/20
1684	stretching vibrations of carbonyl groups C=O in urethane (HDI–BDO)
1483, 1452, 1442, 1421	deforming and scissoring vibrations of CH_2_ groups in both polymer and GPCa
1302, 1261	swinging and bending vibrations of CH_2_ out of the plane groups in polymer and filler with additional C–N stretch at the end
1126, 1131	stretching asymmetric vibrations of C–O–C in ester groups
1040,1080	complex CCCC stretch in branched alkanes with symmetric PO_4_ and C–O–P stretch
959, 939, 882, 835	swinging vibrations of CH_2_ groups in different positions and NH, CH bending deformation vibrations outside of the plane of urethane groups
580,614	deforming vibrations outside of the plane of ester groups with eventual phosphoester and PO_4_ fluctuations

* [39,40,41].

**Table 5:** Contact angle and total surface free energy of obtained PURs and PURs-M.

Symbol	Contact angle (°)			Surface energy (mN/m)		
	Formamide	Ethylene glycol	Water	Acid-part	Base-part	Total surface free energy
PUR	63.6 ± 2	68.2 ± 1	72.1 ± 2	0.04	20.83	31.82
PUR-M	35.8 ± 4	47.5 ± 3	57 ± 3	14.51	21.72	59.09

**Table 6:** The mass loss of the PURs and PURs-M after 15 days of short-term interactions study performed with selected media of the acidic, basic, and oxidative environment.

Sample	Extracted mass (%)		
	5 M NaOH	2 N HCl	0.1 M CoCl_2_/20% H_2_O_2_
**PUR**	50.9 ± 0.2	38.62 ± 0.14	4.25 ± 0.08
**PUR -M**	54.7 ± 0.1	35.81 ± 0.11	2.03 ± 0.12
